# Farrerol alleviates collagenase‐induced tendinopathy by inhibiting ferroptosis in rats

**DOI:** 10.1111/jcmm.17388

**Published:** 2022-05-18

**Authors:** Yongfu Wu, Jun Qian, Kang Li, Wenjun Li, Wenhua Yin, Huaji Jiang

**Affiliations:** ^1^ Department of Pharmacy Yuebei People's Hospital Affiliated to Shantou University Medical College Shaoguan Guangdong China; ^2^ Department of Orthopaedic Spine Surgery Affiliated Hengyang Hospital Southern Medical University (Hengyang Central Hospital) Hengyang Hunan China; ^3^ Department of Orthopaedics General Hospital of Southern Theatre Command Guangzhou Guangdong China; ^4^ Department of Orthopaedics Yuebei People's Hospital Affiliated to Shantou University Medical College Shaoguan Guangdong China; ^5^ 70570 Department of Immunology School of Basic Medical Sciences Southern Medical University Guangzhou Guangdong China

**Keywords:** farrerol, ferroptosis, inflammation, iron accumulation, tendinopathy

## Abstract

Tendinopathy is mainly characterized by local pain, functional limitation and decreased athletic ability, which seriously affects the quality of life of patients and the career of athletes. Farrerol (FA), one of the main active compounds extracted from *Rhododendron* and plants in the *Rhododendron* family, has a wide range of pharmacological activities, such as immunomodulatory, anti‐inflammatory and antiviral effects. However, the effect of FA on tendinopathy is unclear. Here, we investigated the pharmacological effect and mechanism of FA in tendon injury through collagenase‐induced tendinopathy *in vivo* and RSL3‐induced tenocytes injury *in vitro*. The results showed that FA alleviated the infiltration of inflammatory cells, promoted tenogenesis and improved mechanical properties of the Achilles tendon in rats. In addition, ferroptosis inducer RSL3 inhibits the tenogenesis *in vitro* and *in vivo*, which accelerates the progression of tendinopathy. Moreover, FA effectively inhibited iron accumulation and alleviated ferroptosis in the Achilles tendon. Using *in vitro* experiments, we found that FA antagonized ferroptosis by reducing lipid peroxidation and iron accumulation in tenocytes. Finally, we found that glutathione peroxidase 4 silencing could block the protective effect of FA on ferroptosis of tenocytes. Therefore, the results of this study suggest that FA can relieve collagenase‐induced tendinopathy by inhibiting ferroptosis, and reveal that FA may be a potentially effective drug for the treatment of tendinopathy in the future.

## 1 INTRODUCTION

Tendinopathy is characterized by local pain, functional limitation and decreased athletic ability, which seriously affects the quality of life of patients and the career of athletes.^1^, ^2^ Common tendinopathy includes rotator cuff injury, lateral epicondylitis and tendinitis.^3^ The pathogenesis of tendinopathy is still unclear, which seriously limits the development of its treatment. Currently, the treatment of tendinopathy is mainly physical therapy and nonsteroidal anti‐inflammatory drugs (NSAIDs) therapy.^4^, ^5^ However, these treatments can only relieve the symptoms, which cannot completely cure tendinopathy.^3^, ^6^ In addition, NSAIDs can induce gastric ulcers, cardiovascular disease and other adverse complications,^7^ which severely limit their use. Consequently, it is urgent to explore new alternative therapies for tendinopathy.

The pathological features of tendinopathy include disarray of collagen fibres, increased innervation of microvessels and sensory nerves, dysregulation of extracellular matrix homeostasis, immune cells and inflammatory cytokines, as well as increased apoptosis.^8^, ^9^ The traditional view holds that tendinopathy is caused by inflammation; so, it is called "tendinitis." Indeed, there is growing evidence that inflammation plays a key role in the pathogenesis and development of tendinopathy, "tendinopathy" had long been in common usage.^8^, ^10^, ^11^ Inflammation is an indispensable pathological process for the body to self‐repair in response to acute injuries (such as infection and injury).^12^, ^13^ Acute and controlled inflammation have a protective effect on tissue, while chronic and uncontrolled inflammation is harmful. Inflammatory cells mainly include neutrophils, monocytes/macrophages, mast cells and lymphocytes.^14^ Several studies have reported that macrophages, mast cells and T lymphocytes have been detected in acute and chronic tendinopathy.^15^, ^16^, ^17^ Moreover, the degree of inflammatory cell infiltration is associated with the severity of tendinopathy.^18^ Therefore, regulating the inflammatory reaction has a positive therapeutic effect on tendinopathy.

Ferroptosis, a novel iron‐dependent programmed cell death, is mechanistically different from autophagy, apoptosis and necrosis.^19^ The main mechanism of ferroptosis is to catalyse the high expression of unsaturated fatty acids on the cell membrane and induce lipid peroxidation under the action of ferrous iron or ester oxygenase, to induce cell death.^20^, ^21^ In addition, a decrease in glutathione peroxidase 4 (GPX4), the regulatory core enzyme of the antioxidant system (glutathione system), also induces ferroptosis.^22^ It is reported that inflammation plays an important role in promoting ferroptosis.^23^ Therefore, drugs that affect the inflammatory response may also play a critical role in regulating ferroptosis.

Farrerol (FA) is a natural compound with expectorant effects, extracted from *Rhododendron* *dauricum* and other plants in the *Rhododendron* family, which directly influences the respiratory mucosa to promote the movement of cilia and enhance the mechanical removal of foreign bodies in the trachea and bronchus.^24^, ^25^ Clinically, FA is mainly used to treat chronic bronchitis caused by phlegm and viscous mucous.^25^ Meanwhile, FA can also relieve intestinal inflammation by inhibiting the MAPK and NF‐κB pathways.^24^ In addition, FA can reduce the inflammatory response of microglia by regulating the NRF2/ KEAP1 pathway.^26^ Moreover, it was found that FA could be used to treat osteoarthritis, Parkinson's disease and hepatitis.^27^, ^28^, ^29^ However, the effect and mechanism of FA in tendinopathy have not been studied. Therefore, this study aimed to elucidate the pharmacological effect and mechanism of FA in tendinopathy.

## 2 MATERIALS AND METHODS

### 2.1 Reagents

FA was purchased from Yuanye Bio‐Technology. RSL3 and collagenase I was purchased from Sigma. Rat GPX4 siRNA was purchased from Dharmacon. RNAiMax was purchased from Invitrogen. Primary antibodies EGF‐like module‐containing mucin‐like hormone receptor‐like 1/Adhesion G Protein‐Coupled Receptor E1 (F4/80), tenomodulin (TNMD), ferritin heavy chain (FTH), ferritin light chain (FTL), transferrin receptor 1 (TfR1), ferroportin‐1 (FPN1), scleraxis (Scx) and GPX4 were pursued from Abcam. Primary antibodies iNOS, GAPDH, ferroptosis suppressor protein 1 (FSP1), transferrin receptor 1 (TfR1) and secondary antibodies were pursued from ProteinTech.

### 2.2 Animal experiments

Twenty‐four male Sprague‐Dawley rats (8‐weeks old, 200–250 g) were purchased from the Animal Experimental Center of Southern Medical University, China. All experimental procedures were approved by the Animal Ethics Committee of Southern Medical University. The rats were housed under controlled conditions and provided drinking water and food *ad libitum*. After 1 week of adaptation, the rats were randomly divided into four groups: a control group (PBS), collagenase‐induced tendinopathy (CIT) group, CIT+FA group and FA group. As for detecting the relationship between ferroptosis and tendinopathy, rats were randomly divided into two groups: Control group and the RSL3 group. Collagenase‐induced tendinopathy was performed as previously reported.^30^ Briefly, 30 µl of type I collagenase (10 mg/ml) was injected into the right Achilles tendon of rats. One week after injury, the rats were injected with 2 µg/100 μl of FA into the right Achilles tendon. The control group and collagenase group were injected with the same dose of PBS. The RSL3 group was injected with RSL3 (0.1 μM, 100 μl, once a week) into the right Achilles tendon. Four weeks later, the rats were euthanized and their Achilles tendons were collected for further experiments.

### 2.3 Cell culture and treatment

Tenocytes were extracted as previously reported.^31^ Briefly, after the rats were euthanized, the soft tissue was carefully removed around the Achilles tendon. Then, the Achilles tendon was trimmed into 1 × 1 mm tissue fragments using an ophthalmic clipper. Next, 3 mg/ml collagenase type I was added and the tissue was digested in an incubator for 3 h at 37°C. After digestion, the cells were filtered with a 70 mm cell filter and then centrifuged at 300 g for 5 min. The supernatant was discarded, while the cell precipitation was collected. Finally, the collected cells were cultured in low‐glucose DMEM with 10% foetal bovine serum and 100 Umol^−1^ penicillin and 100 Umol^−1^ streptomycins in an incubator at 37°C with 5% CO_2_. After 3 days of cell culture, the medium was changed. When the cells grew to 80% confluency, they were passaged. The passage of three generations of cells was retained for subsequent experimental study. Cells were divided into four groups: control group, RSL3 group, RSL3+FA group and FA group. As for detecting the relationship between ferroptosis and tendinopathy, cells were treated with different concentrations of RSL3 (0–0.1 μM) for 24 h.

### 2.4 Haematoxylin and eosin (H&E) staining

Tendon tissue was fixed in 4% paraformaldehyde (4%) overnight, embedded in paraffin, sectioned (4 μm) and stained with an H&E kit (Beyotime, Shanghai, China) according to the manufacturer's instructions. To analyse the changes in histological scores on H&E stained sections after treatment, the extent of tendon injury was assessed semi‐quantitatively on a scale of 0 to 4 for the percentage of tendon injuries: 0 for 0%–10% injuries, 1 for 10%–25% injuries, 2 for 26%–50%, 3 for 51%–75% and 4 for 76%–100%. At least 6 randomly selected tendon areas were measured on each slide.

### 2.5 Masson staining

The tissue section dewaxing successively put into different concentrations of ethanol (95%, 70% and 30%) and distilled water for 2 min, rinse twice in 30–40℃water for 30–60 s each time and stained with a Masson staining kit (Nanjing Jiancheng BioEngineering‐Ing Institute) according to the manufacturer's instructions.

### 2.6 Immunohistochemical and immunofluorescence staining

Paraffin sections were initially dewaxed and hydrated. For immunohistochemical staining, the sections were incubated with 3% H_2_O_2_ for 15 min at room temperature to quench endogenous peroxidase activity. Sections were then rinsed with distilled water and soaked in PBS for 5 min. Antigen retrieval was performed by putting the sections in a microwave for 1 h. The sections were blocked with 10% goat serum for 1 h at room temperature and incubated with primary antibody iNOS (1:200) overnight at 4°C. The next day, the sections were washed with PBS and incubated with a second antibody (1:2000) for 1 h at room temperature, after which DAB staining was performed. Finally, the sections were viewed under a microscope. For immunofluorescence staining, after the sections were dewaxed and hydrated, they were washed with distilled water and soaked in PBS for 5 min. Antigen retrieval was performed by placing the sections in a microwave. The sections were blocked with 10% goat serum for 1 h at room temperature and incubated with primary antibody F4/80 (1:100) and TNMD(1:2000) overnight at 4°C. Next, fluorescent secondary antibodies were added and incubated for 1 h in darkness. Finally, the sections were sealed and observed under a fluorescence microscope.

### 2.7 RNA extraction and RT‐qPCR

RT‐qPCR was used to detect the expression of transcription factors in each group. RNA was extracted from tissues and cells using Trizol reagent according to the manufacturer's instructions. The SYBR Green RT‐PCR kit (Takara) and ABI Prism were used for qPCR. The PCR amplification reaction was as follows: 95°C for 15 min and 95°C for 10 s for 40 cycles and annealing at 60°C for 35 s. The $2^{‐\Delta \Delta C_{t}}$ method was used to calculate the relative gene expression. The primers used in this study are listed in Table 1.

**TABLE 1:** List of primers used for RT‐qPCR

Gene	Forward(5′−3′)	Reverse(5′−3′)
*Tnmd*	GCGACAATGTGACTATGTAC	GTCTTCTCCACCTTCACTTG
*Mkx*	CCCCGGACATCGGATCTACTA	CTCTTAGGATGAGGATTTAGGTA
*Scx*	CCTTCTGCCTCAGCAACCAG	GGTCCAAAGTGGGGCTCTCCGTGACT
*Mcp1*	CTGTCACGCTTCTGGGCCTGTT	CTTTGGGACACCTGCTGCTGGT
*Pge2*	CTTTAGTCTGGCCACGATGC	ACAGAAGAGCAAGGAGACCC
*Tnfa*	CACCACGCTCTTCTGTCTACTG	GGGCTACGGGCTTGTCACTC
*Il−1b*	GCCCGTCCTCTGTGACTCGT	TGTCGTTGCTTGTCTCTCCTTGTA
*Il−6*	CTGTTGTGGGTGGTATCCTCTGT	TTGCCTTCTTGGGACTGATGTT
*Il−17*	CCATCCATGTGCCTGATGCT	GTTATTGGCCTCGGCGTTTG
*Ftl*	GAGCGTCTCCTCAAGTTG	GGTTCAGGTTCTTCTCCAG
*Fth*	CCAGAACTACCACCAGGACTC	GTTTCTCAGCATGTTCCCTCT
*Fpn1*	GATGGGTCCTTACTGTCTGCTAC	GTCTGCTAATCTGCTCCTGTTTTCT
*Tfr1*	ACTCTGCTTTGCGACTATTGC	TTCTGACTTGTCCGCCTCTT
*Gpx4*	ATACGCTGAGTGTGGTTTGC	CTTCATCCACTTCCACAGCG
*Slc7a11*	ATACGCTGAGTGTGGTTTGC	CTTCATCCACTTCCACAGCG
*Fsp1*	GCGACCTTCAAGGACAACTTCC	GCCAGGATAAGATGTGAGAAGGG
*Gapdh*	AGAAGGCTGGGGCTCATTTG	AGGGGCCATCCACAGTCTTC

### 2.8 Lipid peroxidation assay

The concentration of malondialdehyde (MDA), one of the final products of lipid peroxidation, was determined using a lipid peroxidation colorimetric kit (Nanjing Jiancheng BioEngineering‐Ing Institute). Briefly, tissues or cells were homogenized in frozen RIPA buffer, and the supernatant was collected after centrifugation of 5000 g for 10 min at room temperature. The level of MDA was then determined using a multifunctional microplate analyzer and measuring the absorbance at 532 nm.

### 2.9 Glutathione assay

The concentration of GSH in each treatment group was determined using a GSH kit (Nanjing Jiancheng BioEngineering‐Ing Institute, Nanjing, China). First, tenocytes were seeded into a 96‐well plate at a density of 1 × 10^4^ cells per well. After cell attachment, different stimulus treatments were added according to each experimental group. Finally, the concentration of GSH was determined using a multifunctional microplate analyzer and measuring the absorbance at 412 nm.

### 2.10 Cell viability assay

Cell vitality of each treatment group was determined using the Counting Kit‐8 (CCK‐8) kit. First, tenocytes were seeded in a 96‐well plate at 1 × 10^4^ cells per well. After cell attachment, the cells were incubated with 10 μl CCK‐8 for 2–3 h at 37°C. Finally, the optical density of each well was determined using a multifunctional microplate analyzer and measuring the absorbance at 450 nm.

### 2.11 RNA interference

Cells were transfected with siRNA by reverse transfection using an RNAiMax (Invitrogen) kit according to the manufacturer's instructions.

### 2.12 Biomechanical assay

The tissue specimen was thawed overnight in a refrigerator at 4°C. The soft tissue around the Achilles tendon was removed, the length of the Achilles tendon and the width and thickness of the middle part of the tendon was measured with a digital micrometre, and the specimen was fixed on a uniaxial biomechanical experimental machine. The Achilles tendon was clamped at both ends, and the distance between the clamps was adjusted to make the Achilles tendon extend naturally. Achilles tendon stiffness was calculated from the load‐displacement curve.

### 2.13 Western blotting

Total protein was extracted from fresh tendon tissue or cells with RIPA protein lysate, and a BCA protein kit was used to quantify the protein concentration. After protein denaturation, sodium dodecyl sulfonate‐polyacrylamide gel electrophoresis was carried out. Next, the protein isolate was transferred onto PVDF membranes and blocked in 5% skim milk powder for 2 h at room temperature. The membranes were then incubated with anti‐FTL (1:800), anti‐FTH (1:800), anti‐FPN1 (1:800), anti‐TfR1 (1:800), anti‐GPX4 (1:1000), anti‐SLC7A11 (1:800), anti‐FSP1(1:800), Scx (1:500), TNMD (1:500), and GAPDH (1:3000) overnight at 4 °C, followed by incubation with HRP‐labelled secondary antibody (1:5000) for 1 h at room temperature. The relative protein expression was measured by ImageJ software.

### 2.14 Statistical analysis

The quantitative experimental data were expressed as mean ±standard deviation. SPSS 19.0 software was used for statistical analysis. Statistical significance was determined by one‐way or two‐way analysis of variance (ANOVA). *p* values <0.05 were considered to be statistically significant. At least six animals were used in each group (*n* = 6).

## 3 RESULTS

### 3.1 FA alleviated collagenase‐induced tendinopathy in rats

**FIGURE 1:**
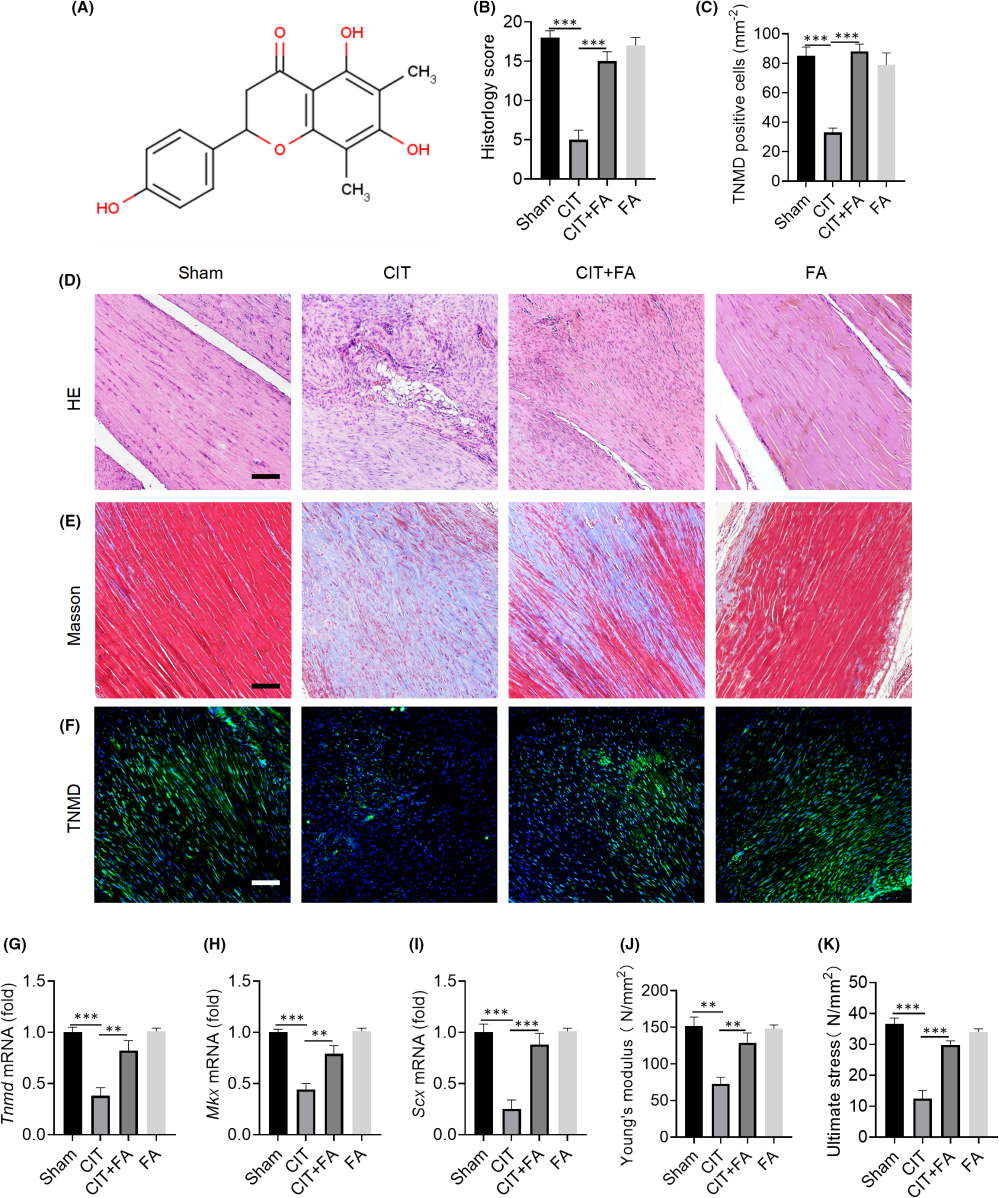
Farrerol improves the biological properties of Achilles tendon in tendinopathy. A: The chemical structural formula of FA. B, D: H&E staining and histological scores were detected after FA treatment for 4 weeks. Scale bar, 100 μm. C, F: Immunofluorescence staining and TNMD‐positive cells were detected count after FA treatment for 4 weeks. Scale bar, 100 μm. E: Masson staining was detected after FA treatment for 4 weeks. Red indicates high stretch stress fibres, while blue indicates low stretch stress fibres. Scale bar, 100 μm. G‐I: The mRNA expression of the tenogenic factors *Tnmd*, *Mkx* and *Scx* was detected after FA treatment for 4 weeks. J: Young's modulus of the Achilles tendon was detected after FA treatment for 4 weeks. K: The maximum tension of the Achilles tendon was detected after FA treatment for 4 weeks. ^**^ *p *< 0.01, ^***^ *p *< 0.001

The chemical structure of FA is shown in Figure 1A. H&E staining showed that the degree of injury in Achilles tendons of the CIT group was more severe and that in the CIT+FA group, the injury was less severe, compared with the sham operation group; however, there was no significant difference between the FA and sham groups (Figure 1B, D). Masson staining results showed that high stretch stress fibres were more obvious in the sham and FA groups, while low stretch stress fibres were more obvious in the CIT group, compared with the CIT+FA group (Figure 1E). Immunofluorescence staining results showed that the expression of the tenogenic protein TNMD was significantly decreased in the CIT group when compared with that in the CIT+FA group (Figure 1C, F). Meanwhile, the mRNA levels of *Tnmd*, *Mkx* and *Scx* declined sharply in the CIT group, while there was a marked increase of these genes in the CIT+FA group (Figure 1G, H, I). In terms of biomechanics, Young's modulus and maximum stretch pressure in the CIT group were significantly lower than that in the CIT+FA group (Figure 1J, K). Therefore, these findings suggest that FA can promote tenogenic differentiation, improve tendon mechanical properties, alleviate tendon injury and relieve the progression of tendinopathy in rats.

### 3.2 FA alleviated the response and infiltration of inflammatory cells

**FIGURE 2:**
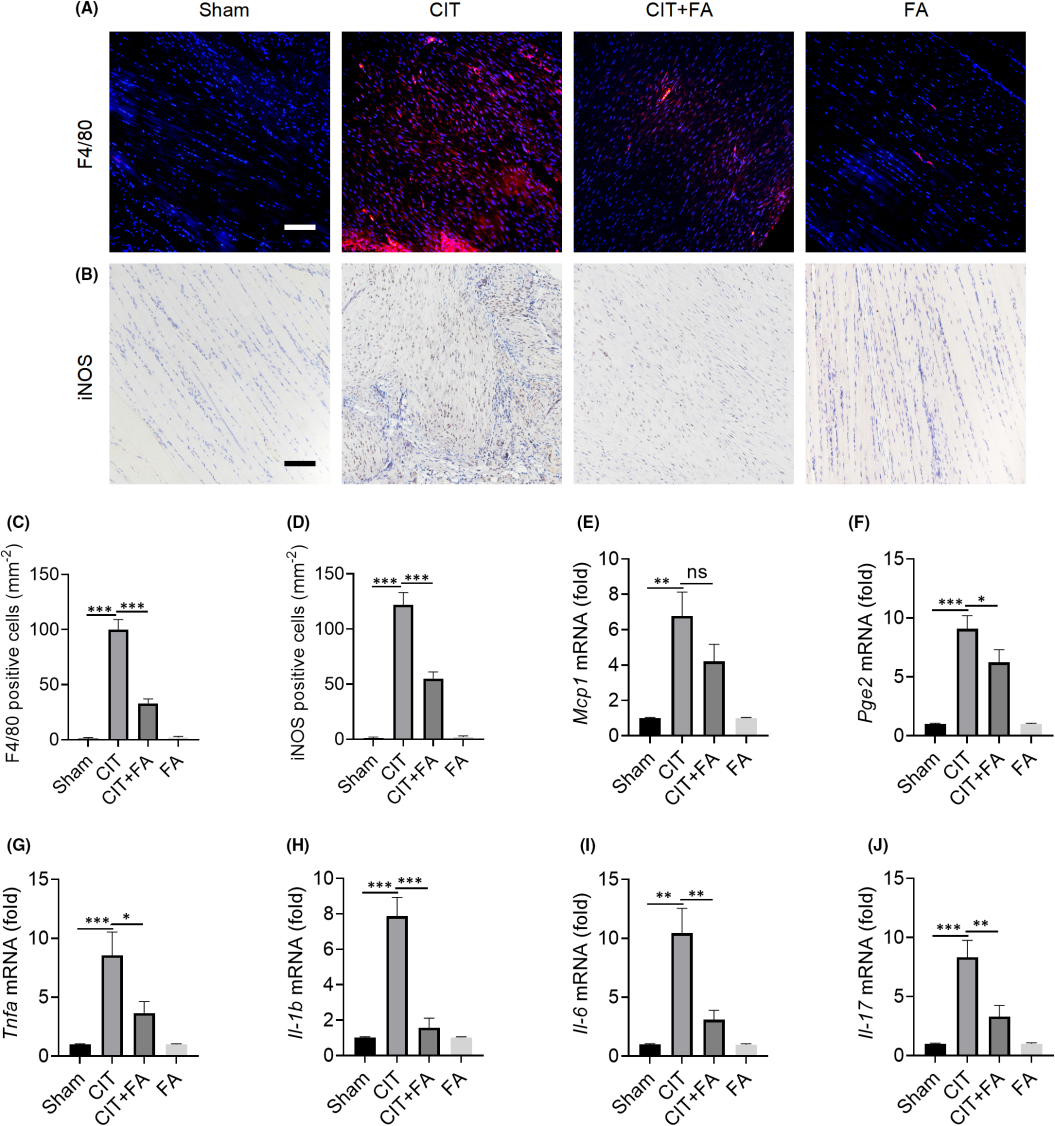
Farrerol alleviates inflammatory cell infiltration in tendinopathy. A, C: Immunofluorescence staining was detected, and F4/80‐positive cells were counted after FA treatment for 4 weeks. Scale bar, 100 μm. B, D: Immunohistochemical staining and iNOS‐positive cells were counted after treatment for 4 weeks. Scale bar, 100 μm. E–J: The mRNA expression of the inflammatory cytokines *Mcp1*, *Pge2*, *Tnfa*, *Il*‐*1b*, *Il*‐*17* and *Il*‐*6*. ^*^ *p *< 0.05, ^**^ *p *< 0.01, ^***^ *p *< 0.001

Next, we further investigated the effects of FA on the inflammatory response and inflammatory cell infiltration after tendon injury in rats. Previous studies showed that macrophages are the key immune cells in the early inflammation of tendinopathy. Here, we detected the infiltration of F4/80‐positive cells. We found that F4/80‐positive cells were rarely detected in the sham and FA groups, but were significantly increased in the CIT group. Furthermore, macrophage infiltration was significantly reduced in the CIT+FA group when compared with the CIT group (Figure 2A, C). It was also found that iNOS, a marker of M1‐type macrophages, was increased in the CIT group. As expected, FA reduced the expression of iNOS, thus alleviating the inflammatory response (Figure 2B, D). In addition, we found that the mRNA levels of the pro‐inflammatory cytokines *Mcp1*, *Pge2*, *Tnfa*, *Il*‐*1b*, *Il*‐*6* and *Il*‐*17* were significantly increased in the CIT group, while the expression of these pro‐inflammatory cytokines was significantly inhibited in the CIT+FA group (Figure 2E–J). Therefore, the results indicated that FA effectively inhibited the inflammatory response in collagenase‐induced tendinopathy.

### 3.3 Ferroptosis promoted the development of tendinopathy

**FIGURE 3:**
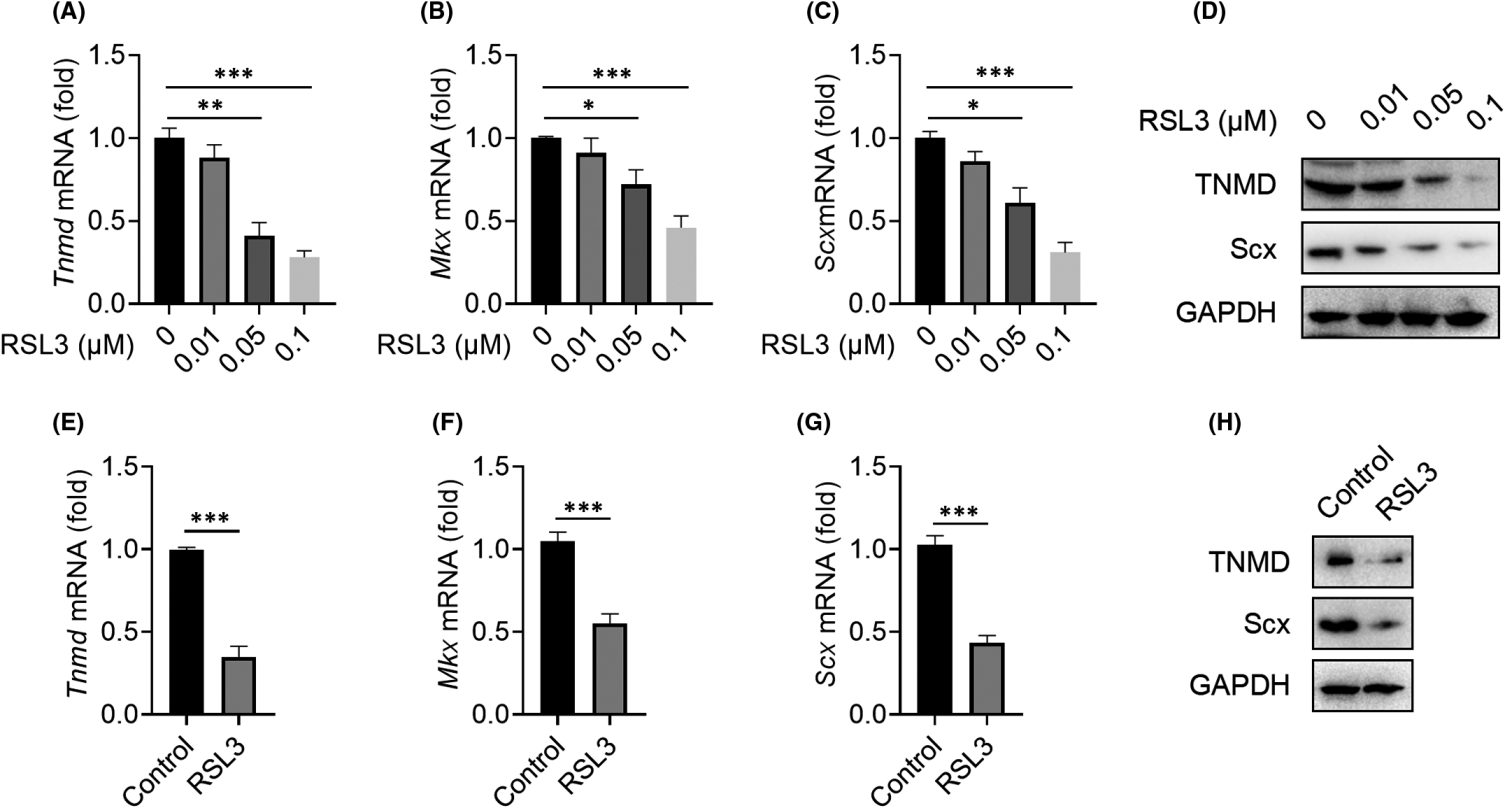
Ferroptosis promoted the progress of tendinopathy. A–D: Tenocytes were treated with RSL3 (0–0.1 μM) for 24 h, and then, cellular RNA and protein were collected for qPCR and WB detection. E–H: RSL3 (0.1 μM, 100 μl, once a week) was injected locally into the Achilles tendon tissue of rats with tendinopathy, and the Achilles tendon was collected for qPCR and WB after 4 weeks.^**^ *p *< 0.01, ^***^ *p *< 0.001

Inflammation has been reported to accelerate ferroptosis.^32^, ^33^, ^34^ However, the role of ferroptosis in tendinopathy is still unclear. RSL3 is a widely used ferroptosis inducer. Tenocytes were incubated with indicated concentrations of RSL3 for 24 h. As shown in Figure 3A‐C, after inducing ferroptosis with RSL3, the mRNA expressions of *Tnmd*, *Mkx* and *Scx* were decreased in a dose‐dependent manner. Likewise, WB also obtained consistent results (Figure 3D). In vivo, we found that RSL3 significantly reduced the expression of tenogenic genes (*Tnmd*, *Mkx* and *Scx*) and tenogenic proteins (TNMD and Scx) (Figure 3E‐H). Therefore, ferroptosis inhibited the tenogenesis and accelerated the progression of tendinopathy in vitro and in vivo.

### 3.4 FA alleviated iron accumulation in collagenase‐induced tendinopathy of rats

**FIGURE 4:**
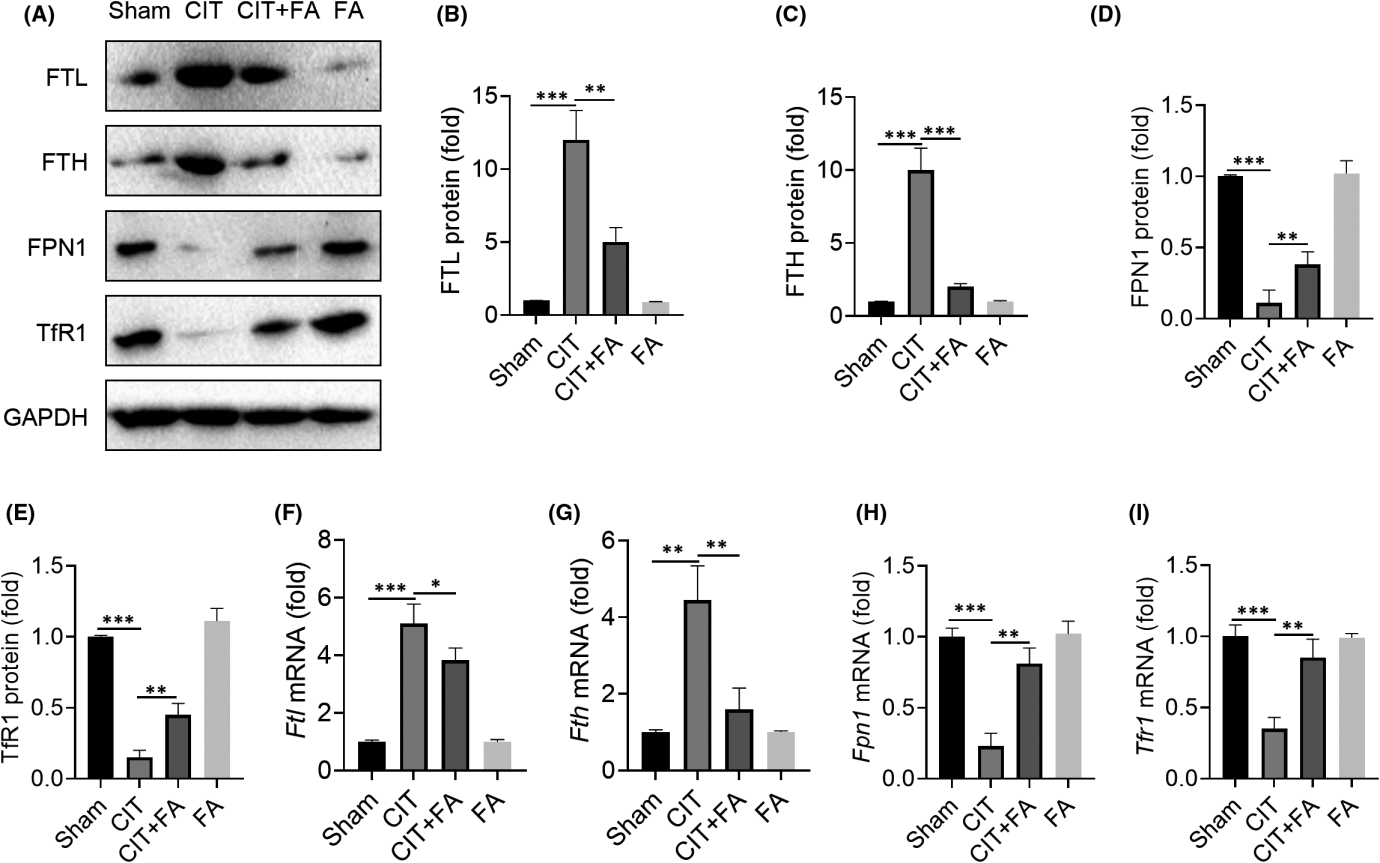
Farrerol inhibits iron accumulation in tendinopathy. A–E: Western blot results of the expression of iron‐related proteins FTL, FTH, FPN1 and TfR1 after FA treatment for 4 weeks. F‐I: FA inhibits the expression of the iron accumulation‐related genes *Ftl*, *Fth*, *Fpn1* and *Tfr1*. ^*^ *p *< 0.05, ^**^ *p *< 0.01, ^***^ *p *< 0.001

Iron accumulation is vital in the initiation of ferroptosis. Iron homeostasis in the body is closely regulated by iron metabolism‐related proteins, which regulate the transport of iron and prevent excessive iron accumulation in cells. Next, we investigated the expression of iron accumulation‐related proteins in collagenase‐induced tendinopathy. The iron output protein FPN1 was significantly reduced in the CIT group. However, FA significantly increased the expression of FPN1 protein in the CIT+FA group (Figure 4A, D). The expression of TfR1 was decreased in the CIT group, while it was effectively restored in the CIT+FA group (Figure 4A, E). The expressions of FTL and FTH were up‐regulated in the CIT group. However, FTL and FTH in the CIT+FA group were significantly higher than that in the CIT group (Figure 3A, B, C). In addition, FA also inhibited the mRNA levels of *Ftl*, *Fth*, *Fpn1* and *Tfr1* (Figure 4F‐I). Therefore, these results indicated that FA alleviated iron accumulation in collagenase‐induced tendinopathy of rats.

### 3.5 FA ameliorated collagenase‐induced tendinopathy in rats by targeting ferroptosis

**FIGURE 5:**
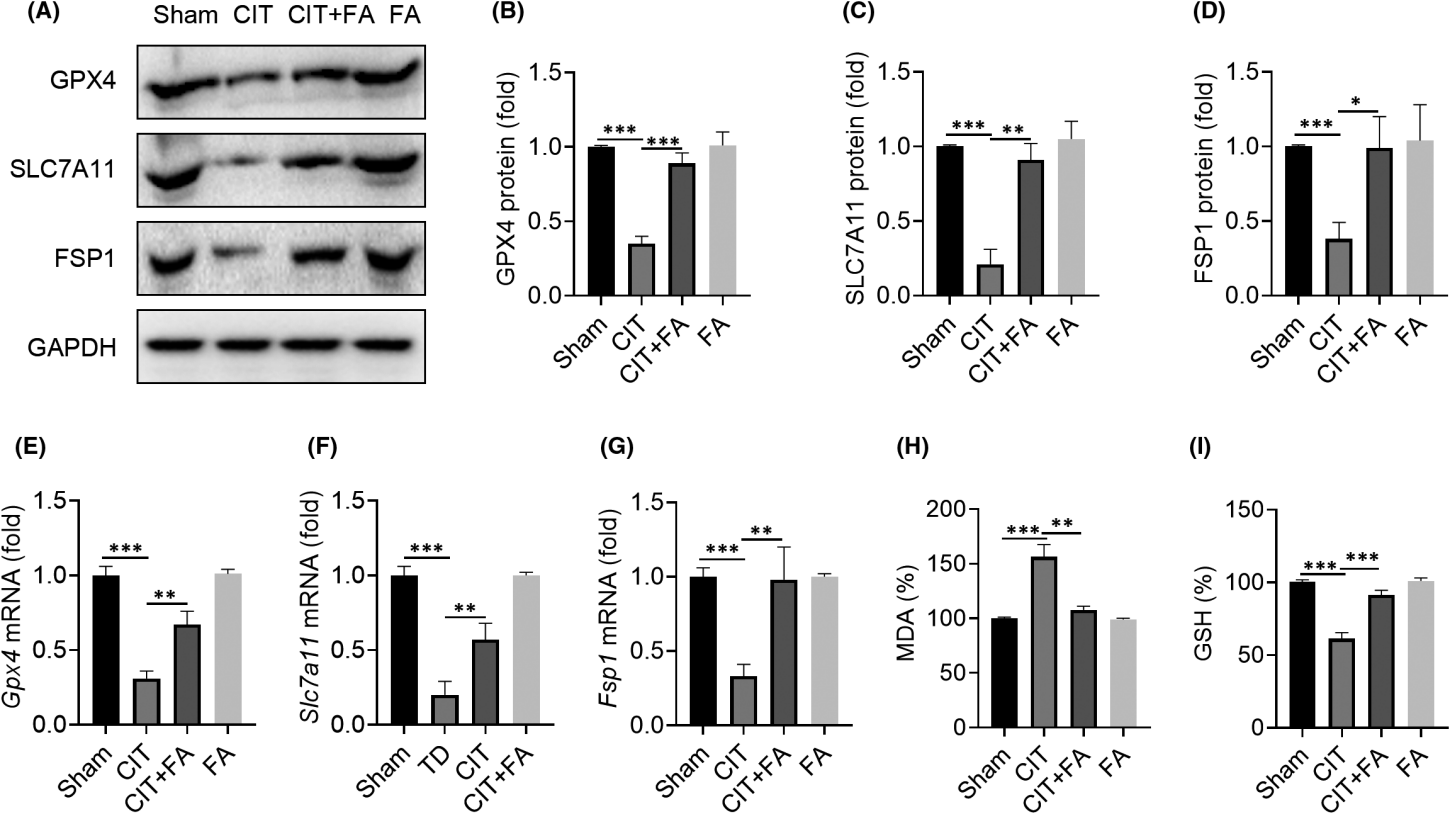
Farrerol alleviates ferroptosis in tendinopathy. A–D: Western blot results of the expression of the ferroptosis‐related proteins GPX4, SLC7A11 and FSP1 after FA treatment for 4 weeks. E–G: qPCR results of the mRNA expression of *Gpx4*, *Slc7a11* and *Fsp1* after FA treatment for 4 weeks. H: Detection of the effect of FA on the levels of MDA in tendinopathy. I: Detection of the effect of FA on GSH levels in tendinopathy. ^*^ *p *< 0.05, ^**^ *p *< 0.01, ^***^ *p *< 0.001

GPX4, SLC7A11 and FSP1 are important biomarkers of ferroptosis. By examining the expression of these biomarkers, we further elucidated the therapeutic effect and mechanism of FA in regulating ferroptosis in collagenase‐induced tendinopathy. As shown in Figure 5A–D, the protein expression of GPX4, SLC7A11 and FSP1 was significantly reduced in the CIT group. However, treatment with FA effectively reversed the reduction of these proteins. In addition, FA also promoted the mRNA expression of *Fsp1*, *Slc7a11* and *Gpx4* in the CIT+FA group (Figure 5E–G). A significantly increased concentration of MDA was found in the CIT group, while the production of MDA was inhibited by FA treatment (Figure 5H). These data indicate that FA could inhibit lipid peroxidation. Furthermore, GSH levels were decreased in the CIT group but were restored in the CIT+FA group (Figure 5I). These data showed that FA could prevent the progression of collagenase‐induced tendinopathy in rats by inhibiting ferroptosis and alleviating lipid peroxidation.

### 3.6 FA relieved ferroptosis in vitro

**FIGURE 6:**
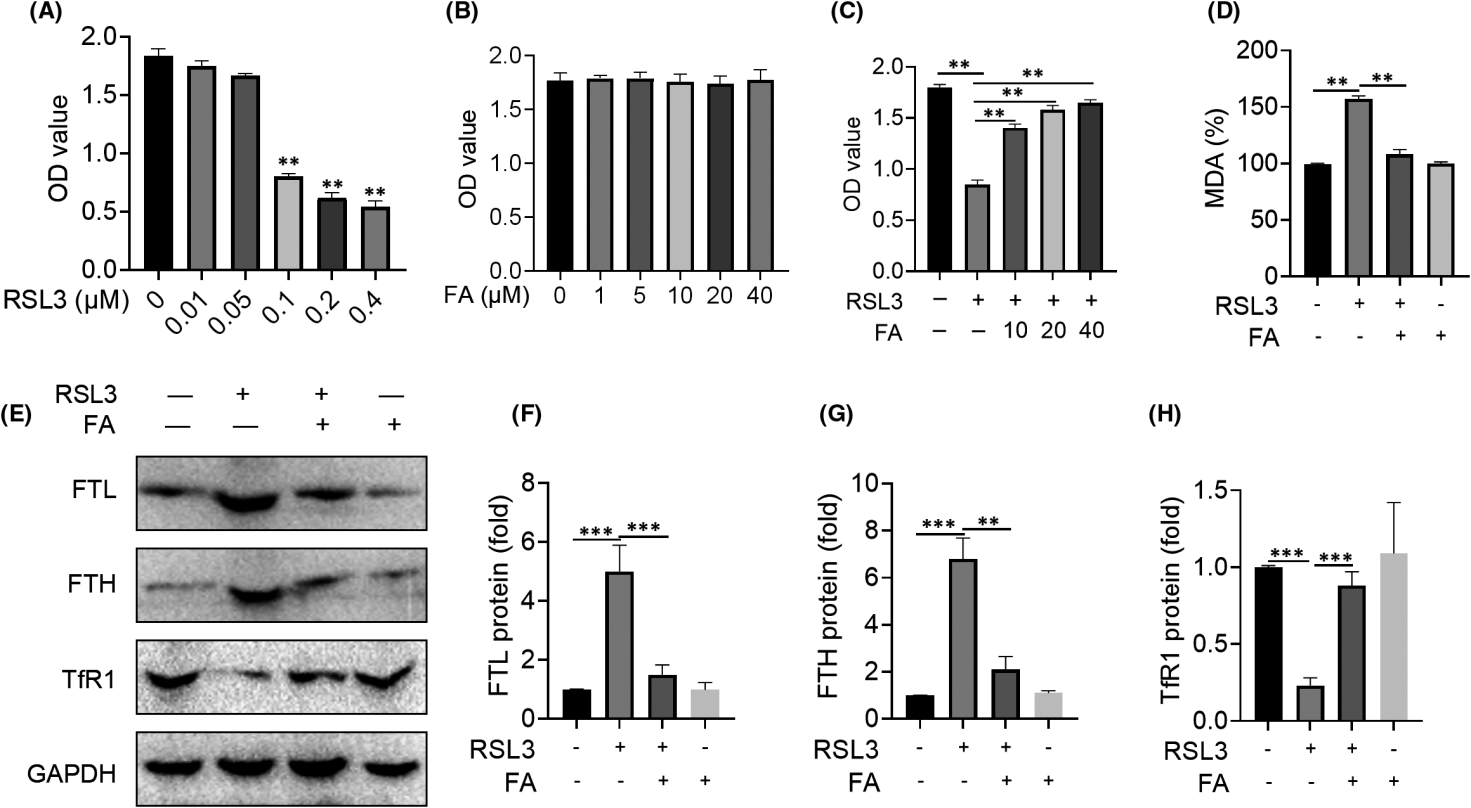
Farrerol protects tenocytes from ferroptosis. A: CCK‐8 assay results of RSL3 (0–0.4 μM) on the viability of tenocytes. B: CCK‐8 assay results of FA (0–40 μM) on the viability of tenocytes. C: CCK‐8 assay was used to detect the effect of FA (0–40 μM) on ferroptosis induced by RSL3 in tenocytes. D: The effect of FA (40 μM) on the expression of MDA induced by RSL3(0.1 μM)in tenocytes. E‐H: Western blot results of FA (40 μM) treatment for 24 h on the iron accumulation‐related proteins FTL, FTH and TFR induced by RSL3(0.1 μM) in tenocytes. ^**^ *p *< 0.01, ^***^ *p *< 0.001

RSL3 is a classic ferroptosis inducer. We treated tenocytes with different concentrations of RSL3 for 24 h to determine its effect on cell viability. After treatment with RSL3, the cell viability decreased in a dose‐dependent manner, and the cell viability decreased by nearly half at 0.1 μM (Figure 6A). Therefore, 0.1 μM RSL3 was deemed to be an effective concentration to induce ferroptosis in tenocytes. To verify if FA was toxic to tenocytes, we added different concentrations of FA (1, 5, 10, 20 and 40 μM) to cells for 24 h. We found that FA had no cytotoxic effect on tenocytes at these concentrations (Figure 6B). Next, to test the effect of FA on ferroptosis in tenocytes, we used different concentrations of FA (10, 20 and 40 μM) to treat cells for 24 h under the condition of RSL3 (0.1 μM). The results showed that the cell viability was increased in a concentration‐dependent manner after treatment with different concentrations of FA (Figure 6C). Meanwhile, we also found that MDA levels were significantly increased after RSL3 treatment, while the increase of MDA levels was inhibited after FA treatment (Figure 6D). In addition, we also detected the protein expression of FTL, FTH and TfR1 in tenocytes in each treatment group. It was found that the protein expression of FTH and FTL increased after RSL3 treatment, while the protein expression of TfR1 decreased. After treatment with FA, the increase in the expression of FTL and FTH was inhibited, whereas the expression of TfR1 protein was increased (Figure 6E–H). Therefore, these results suggest that FA relieved ferroptosis induced by RSL3.

### 3.7 FA increased the expression of the ferroptosis‐related protein GPX4

**FIGURE 7:**
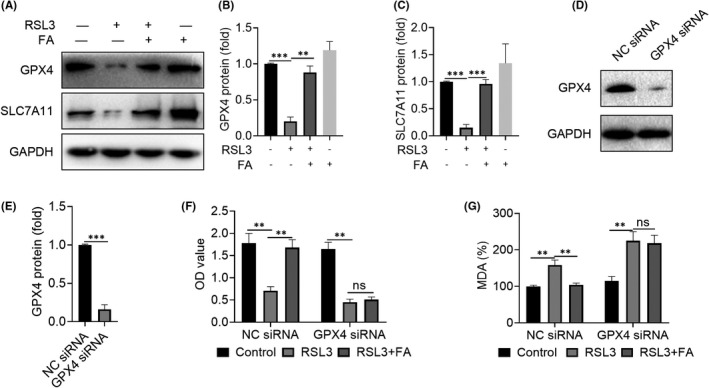
Farrerol plays an anti‐ferroptosis role through a GPX4‐dependent pathway. A–C: Western blot results of the effect of FA(40 μM) on the expression of GPX4 and SLC7A11 induced by RSL3 (0.1 μM)in tenocytes. D–E: The silencing efficiency of GXP4 siRNA. F: CCK‐8 assay results of the effect of FA (40 μM) on ferroptosis in tenocytes after silencing GPX4. G: After GPX4 silencing, the effects of FA (40 μM) on MDA levels in RSL3‐treated tenocytes were detected. ^**^ *p *< 0.01, ^***^ *p *< 0.001

We then examined the effects of FA on GPX4 and SLC7A11 expression *in vitro*. The results showed that RSL3 decreased the expression of GPX4 and SLC7A11 in tenocytes, while FA restored their expression (Figure 7A–C). Next, we used siRNA to knockdown endogenous GPX4 to evaluate the potential role of GPX4 in FA‐mediated anti‐ferroptosis. As shown in Figure 7D and E, GPX4 siRNA could effectively silence GPX4 expression with a silencing efficiency of about 80%. The use of FA after GPX4 silencing did not reverse ferroptosis induced by RSL3 (Figure 7F). In addition, the use of FA after GPX4 silencing did not reduce the increase in MDA induced by RSL3 (Figure 7G). Taken together, the data indicate that FA inhibits ferroptosis in tenocytes through a GPX4‐dependent pathway, thus effectively alleviating the progression of tendinopathy.

## 4 DISCUSSION

FA is an expectorant drug derived from Rhododendron dauricum and other Rhododendron plants.^26^ FA has a wide range of pharmacological effects, such as anti‐inflammatory, antibacterial, antioxidant and other biological activities.^35^ Here, we found that FA has a good therapeutic effect on tendinopathy. On the one hand, FA alleviated tendon injury induced by collagenase in rats. On the other hand, FA alleviated the death of tenocytes induced by RSL3. Specifically, the protective effect of FA on tendinopathy is mainly reflected in optimizing the tendon histological structure, improving tendon biomechanical properties, reducing the inflammatory response of the tendon tissue and decreasing the infiltration of inflammatory cells in the Achilles tendon. We also found that ferroptosis promoted the progress of tendinopathy. At the molecular level, FA reduces iron accumulation and inhibits ferroptosis in tenocytes via a GPX4‐dependent pathway. Furthermore, the use of FA alone in this study did not cause significant adverse reactions in rats, and the use of FA at the maximum concentration did not cause a decrease in the activity of tenocytes. These results indicate that FA has good bioactivity and safety, and can effectively inhibit the progression of tendinopathy. Therefore, FA may be a potential drug for the treatment of tendinopathy.

Iron is an essential element that is filtered through the glomeruli and reabsorbed in the renal tubules.^36^ The body's iron metabolism involves iron intake, storage, utilization and excretion. Meanwhile, it is regulated by a variety of proteins.^37^ After dietary intake, iron binds to transferrin.^38^ Transferrin‐bound iron then binds to the TfR1 receptor on the plasma membrane and is transmitted to numerous tissues through the blood circulation.^39^ Excess iron is stored in ferritin storage proteins encoded by ferritin light chain and ferritin heavy chain 1 for iron utilization. FPN1/IREG1 is a kind of iron outflow pump existing in vertebrates, and the intracellular iron is mainly exported through FPN1/IREG1.^39^ Abnormalities in iron homeostasis are associated with a variety of diseases.^40^ In collagenase‐induced tendinopathy, we found a significant increase in iron concentration in the tendons. Furthermore, we found that tendinopathy was related to ferroptosis, which played an important role in the pathogenesis of tendinopathy. Iron is necessary for ferroptosis and iron‐chelating agents have been shown to prevent or reduce ferroptosis. In our experiment, we found that FA inhibited iron accumulation in collagen‐induced tendinopathy in rats, as well as iron accumulation in tenocytes induced by RSL3. Furthermore, we found that the expression of FTH and FTL increased in tendinopathy, while the expression of TfR1 decreased; a trend that was reversed after FA treatment. Therefore, these results suggest that FA can be used as a novel ferroptosis inhibitor in the treatment of tendinopathy. In addition, FA is a potential treatment for other iron‐related diseases.

The main mechanisms of ferroptosis involve several mechanisms: (1) GPX4 inactivation due to GSH consumption: As previously mentioned, GPX4 is the only GPX involved in liposome peroxide reduction in cells. Specifically, GPX4 alters the peroxide bonding strength to the hydroxyl group in lipid peroxide, which causes a reduction in its activity. The main targets of GPX4 include system Xc, glutamate‐cysteine ligase and glutathione S‐transferase.^41^ (2) GPX4 inactivation: In addition to indirectly acting on GSH, which activates GPX4, GPX4 can also be directly inactivated with GPX4 inhibitors including squalene synthase and HMG‐CoA reductase. (3) Iron ion input and iron ion reduction: Iron accumulates in the cell in the form of ferric iron, which can initiate liposome peroxidation through the Fenton reaction.^23^ GPX4 is an antioxidant defense enzyme that can repair oxidative damage to lipids and is a central regulator of ferritin inactivation.^22^ Based on our research, FA inhibited the expression of ferroptosis‐related proteins such as FSP1, SLC7A11 and GPX4. In addition, after GPX4 silencing, FA did not affect ferroptosis, suggesting that FA exerted its anti‐ferroptosis function through a GPX4‐dependent pathway. Therefore, regulation of GPX4 may be important for the prevention and treatment of tendinopathy and the promotion of tendon healing.

Although this study has shown FA as a potential drug for the treatment of tendinopathy, there were still several limitations to the current study. First, due to ethical problems, only rat tenocytes were used in experiments. However, whether the same effect of FA on human tenocytes exists is worth further study. Second, no positive control was used, and a comparison of the efficacy of FA with other tendinopathy drugs, such as NSAIDs, will need to be further investigated in future experiments.

In summary, FA can inhibit the ferroptosis of tenocytes, improve the biological properties of tendons and promote tendon healing, thereby effectively alleviating the progression of tendinopathy. Therefore, FA is a new potential drug for the treatment of tendinopathy and iron‐related diseases. However, further clinical trials are needed to demonstrate its efficacy and safety in human tendinopathy.

## AUTHOR CONTRIBUTIONS

**Yongfu Wu:** Data curation (equal); Formal analysis (equal); Investigation (equal); Software (equal); Writing – original draft (equal). **Jun Qian:** Data curation (equal); Formal analysis (equal); Investigation (equal); Writing – original draft (equal). **Kang Li:** Data curation (equal); Formal analysis (equal); Investigation (equal); Software (equal); Writing – original draft (equal). **Wenjun Li:** Formal analysis (equal); Software (equal). **Wenhua Yin:** Conceptualization (equal); Data curation (equal); Formal analysis (equal); Investigation (equal); Methodology (equal); Project administration (equal); Resources (equal); Software (equal); Supervision (equal); Validation (equal); Visualization (equal); Writing – review & editing (equal). **Huaji Jiang:** Conceptualization (equal); Data curation (equal); Formal analysis (equal); Funding acquisition (equal); Investigation (equal); Methodology (equal); Project administration (equal); Resources (equal); Software (equal); Supervision (equal); Validation (equal); Visualization (equal); Writing – review & editing (equal).

## CONFLICT OF INTEREST

The authors confirm that there are no conflicts of interest.

## Data Availability

The original contributions presented in the study are included in the article/supplementary material, further inquiries can be directed to the corresponding author.

## References

[1] Rowe V , Hemmings S , Barton C , Malliaras P , Maffulli N , Morrissey D . Conservative management of midportion achilles tendinopathy. Sports Med. 2012;42(11):941‐967. 23006143 10.1007/BF03262305

[2] Bannuru RR , Flavin NE , Vaysbrot E , Harvey W , McAlindon T . High‐energy extracorporeal shock‐wave therapy for treating chronic calcific tendinitis of the shoulder: a systematic review. Ann Intern Med. 2014;160(8):542‐549. 24733195 10.7326/M13-1982

[3] Wilson JJ , Best TM . Common overuse tendon problems: a review and recommendations for treatment. Am Fam Physician. 2005;72(5):811‐818. 16156339

[4] Aicale R , Bisaccia RD , Oliviero A , Oliva F , Maffulli N . Current pharmacological approaches to the treatment of tendinopathy. Expert Opin Pharmacother. 2020;21(12):1467‐1477. doi:10.1080/14656566.2020.1763306 32511031

[5] Calfee RP , Patel A , DaSilva MF , Akelman E . Management of lateral epicondylitis: current concepts. J Am Acad Orthop Surg. 2008;16(1):19‐29. doi:10.5435/00124635-200801000-00004 18180389

[6] Di Matteo B , Filardo G , Kon E , Marcacci M . Platelet‐rich plasma: evidence for the treatment of patellar and Achilles tendinopathy—a systematic review. Musculoskelet Surg. 2015;99(1):1‐9. 25323041 10.1007/s12306-014-0340-1

[7] Vonkeman HE , van de Laar MAFJ . Nonsteroidal anti‐inflammatory drugs: adverse effects and their prevention. Semin Arthritis Rheum. 2010;39(4):294‐312 18823646 10.1016/j.semarthrit.2008.08.001

[8] Millar NL , Silbernagel KG , Thorborg K , et al. Tendinopathy. Nat Rev Dis Primers. 2021;7(1):1. doi:10.1038/s41572-020-00234-1 33414454

[9] Carlsson O , Schizas N , Li J , Ackermann PW . Substance P injections enhance tissue proliferation and regulate sensory nerve ingrowth in rat tendon repair. Nat Rev Dis Primers. 2011;21(4):562‐569. doi:10.1111/j.1600-0838.2009.01080.x 20459473

[10] D'Addona A , Maffulli N , Formisano S , Rosa D . Inflammation in tendinopathy. Surgeon. 2017;15(5):297‐302. 28596062 10.1016/j.surge.2017.04.004

[11] Millar NL , Murrell GAC , McInnes IB . Inflammatory mechanisms in tendinopathy – towards translation. Nat Rev Rheumatol. 2017;13(2):110‐122. doi:10.1038/nrrheum.2016.213 28119539

[12] Tidball JG . Inflammatory cell response to acute muscle injury. Med Sci Sports Exerc. 1995;27(7):1022‐1032. 7564969 10.1249/00005768-199507000-00011

[13] Lucas SM , Rothwell NJ , Gibson RM . The role of inflammation in CNS injury and disease. Br J Pharmacol. 2006;147(S1):S232‐S240. 16402109 10.1038/sj.bjp.0706400 PMC1760754

[14] Ribatti D , Crivellato E . Immune cells and angiogenesis. J Cell Mol Med. 2009;13(9a):2822‐2833. 19538473 10.1111/j.1582-4934.2009.00810.x PMC4498938

[15] Jomaa G , Kwan C‐K , Fu S‐C , et al. A systematic review of inflammatory cells and markers in human tendinopathy. BMC Musculoskelet Disord. 2020;21(1):1‐13. 10.1186/s12891-020-3094-y PMC7006114 32028937

[16] Dean BJF , Gettings P , Dakin SG , Carr AJ . Are inflammatory cells increased in painful human tendinopathy? A systematic review. Br J Sports Med. 2016;50(4):216‐220. 26246419 10.1136/bjsports-2015-094754

[17] Sunwoo JY , Eliasberg CD , Carballo CB , Rodeo SA . The role of the macrophage in tendinopathy and tendon healing. J Orthop Res. 2020;38(8):1666‐1675. 32190920 10.1002/jor.24667

[18] Kaux J‐F , Forthomme B , Goff CL , Crielaard J‐M , Croisier J‐L . Current opinions on tendinopathy. J Sports Sci Med. 2011;10(2):238‐253. 24149868 PMC3761855

[19] Dixon Scott J , Lemberg Kathryn M , Lamprecht Michael R , et al. Ferroptosis: an iron‐dependent form of nonapoptotic cell death. Cell. 2012;149(5):1060‐1072. doi:10.1016/j.cell.2012.03.04 22632970 PMC3367386

[20] Reichert CO , de Freitas FA , Sampaio‐Silva J , et al. Ferroptosis mechanisms involved in neurodegenerative diseases. Int J Mol Sci. 2020;21(22):8765. 10.3390/ijms21228765 PMC7699575 33233496

[21] Zhou B , Liu J , Kang R , Klionsky DJ , Kroemer G , Tang D . Ferroptosis is a type of autophagy‐dependent cell death. Semin Cancer Biol. 2020;66:89‐100. doi:10.1016/j.semcancer.2019.03.002 30880243

[22] Imai H , Matsuoka M , Kumagai T , Sakamoto T , Koumura T . Lipid peroxidation‐dependent cell death regulated by GPx4 and ferroptosis. Apoptotic and Non‐apoptotic Cell Death. 2016;143‐170. 10.1007/82_2016_508 28204974

[23] Tang D , Kroemer G . Ferroptosis. Curr Biol. 2020;30(21):R1292‐R1297. 33142092 10.1016/j.cub.2020.09.068

[24] Li Y , Gong Q , Guo W , et al. Farrerol relieve lipopolysaccharide (LPS)‐induced mastitis by inhibiting AKT/NF‐κB p65, ERK1/2 and P38 signaling pathway. Int J Mol Sci. 2018;19(6):1770. 10.3390/ijms19061770 PMC6032361 29904013

[25] Ci X , Chu X , Wei M , Yang X , Cai Q , Deng X . Different effects of farrerol on an OVA‐induced allergic asthma and LPS‐induced acute lung injury. PLoS One. 2012;7(4):e34634. 22563373 10.1371/journal.pone.0034634 PMC3338508

[26] Cui B , Zhang S , Wang Y , Guo Y . Farrerol attenuates β‐amyloid‐induced oxidative stress and inflammation through Nrf2/Keap1 pathway in a microglia cell line. Biomed Pharmacother. 2019;109:112‐119. 30396067 10.1016/j.biopha.2018.10.053

[27] Zhang H , Yan J , Zhuang Y , Han G . Anti‐inflammatory effects of farrerol on IL‐1β‐stimulated human osteoarthritis chondrocytes. Eur J Pharmacol. 2015;764:443‐447. 26162701 10.1016/j.ejphar.2015.07.012

[28] Li Y , Zeng Y , Meng T , et al. Farrerol protects dopaminergic neurons in a rat model of lipopolysaccharide‐induced Parkinson's disease by suppressing the activation of the AKT and NF‐κB signaling pathways. Int Immunopharmacol. 2019;75:105739. 31351366 10.1016/j.intimp.2019.105739

[29] Sharma S , Bhatia V . Phytochemicals for drug discovery in Alzheimer’s disease: in silico advances. Curr Pharm Des. 2021;27(25):2848‐2860. 32988343 10.2174/1381612826666200928161721

[30] Wang Y , He G , Tang H , et al. Aspirin promotes tenogenic differentiation of tendon stem cells and facilitates tendinopathy healing through regulating the GDF7/Smad1/5 signaling pathway. J Cell Physiol. 2020;235(5):4778‐4789. 31637734 10.1002/jcp.29355

[31] Abraham AC , Shah SA , Golman M , et al. Targeting the NF‐κB signaling pathway in chronic tendon disease. Sci Transl Med. 2019;11(481): eaav4319. doi:10.1126/scitranslmed.aav4319 30814338 PMC6534967

[32] Wang F , He J , Xing R , Sha T , Sun B . Molecular mechanisms of ferroptosis and their role in inflammation. Int Rev Immunol. 2021;1‐11. doi:10.1080/08830185.2021.2016739 34918993

[33] Chen X , Kang R , Kroemer G , Tang D . Ferroptosis in infection, inflammation, and immunity. J Exp Med. 2021;218(6):e20210518. doi:10.1084/jem.20210518 33978684 PMC8126980

[34] Sun Y , Chen P , Zhai B , et al. The emerging role of ferroptosis in inflammation. Biomed Pharmacother. 2020;127:110108. doi:10.1016/j.biopha.2020.110108 32234642

[35] Ran X , Li Y , Chen G , et al. Farrerol ameliorates TNBS‐induced colonic inflammation by inhibiting ERK1/2, JNK1/2, and NF‐κB signaling pathway. Int J Mol Sci. 2018;19(7):2037. 10.3390/ijms19072037 PMC6073308 30011811

[36] Shah SV . Oxidants and iron in chronic kidney disease. Kidney Int. 2004;66:S50‐S55. 10.1111/j.1523-1755.2004.09108.x 15461704

[37] Wang J , Pantopoulos K . Regulation of cellular iron metabolism. Biochem J. 2011;434(3):365‐381. 21348856 10.1042/BJ20101825 PMC3048577

[38] Andrews NC . Disorders of iron metabolism. N Engl J Med. 1999;341(26):1986‐1995. 10607817 10.1056/NEJM199912233412607

[39] von Drygalski A , Adamson JW . Iron metabolism in man. J Parenter Enteral Nutr. 2013;37(5):599‐606. 10.1177/0148607112459648 22968710

[40] Pantopoulos K , Porwal SK , Tartakoff A , Devireddy L . Mechanisms of mammalian iron homeostasis. Biochemistry. 2012;51(29):5705‐5724. 22703180 10.1021/bi300752r PMC3572738

[41] Ursini F , Maiorino M . Lipid peroxidation and ferroptosis: The role of GSH and GPx4. Free Radic Biol Med. 2020;152:175‐185. 32165281 10.1016/j.freeradbiomed.2020.02.027

